# Experimental Determination of Silicon Isotope Fractionation in Rice

**DOI:** 10.1371/journal.pone.0168970

**Published:** 2016-12-30

**Authors:** Yan Sun, Liang-huan Wu, Xiao-yan Li

**Affiliations:** 1 Ministry of Education Key Laboratory of Environmental Remediation and Ecosystem Health, College of Environmental and Resource Sciences, Zhejiang University, Hangzhou, China; 2 Zhejiang Provincial Key Laboratory of Subtropical Soil and Plant Nutrition, College of Environmental and Resource Sciences, Zhejiang University, Hangzhou, China; Universidade do Minho, PORTUGAL

## Abstract

Analyzing variations in silicon (Si) isotopes can help elucidate the biogeochemical Si cycle and Si accumulation processes of higher plants. Importantly, the composition of Si isotopes in higher plants has yet to be studied comprehensively and our knowledge of the distribution of Si isotopes in higher plants lags behind that of Si isotopes in marine organisms, such as diatoms. In the present study, we investigated the isotope fractionation that occurs during the uptake and transport of Si in rice, using a series of hydroponic experiments with different external concentrations of Si. We found that an active mechanism was responsible for the majority of Si uptake and transport at lower Si levels and that the uptake of Si by rice roots was significantly suppressed by both low temperature and metabolic inhibitors. In addition, light Si isotopes (^28^Si) entered roots more readily than heavy Si isotopes (^30^Si) when the active mechanism was inhibited. Therefore, we conclude that biologically mediated isotope fractionation occurs during the uptake of Si by rice roots. In addition, both active and passive Si uptake components co-exist in rice, and the fractionation effect is enhanced when more Si is absorbed by plants.

## Introduction

As the second most mass-abundant element on the Earth’s crust (after oxygen) [1], the biogeochemistry of silicon (Si) has attracted steadily growing scientific interest. The element is essential for diatom growth [1], and researchers have demonstrated that phytoplankton preferentially take up lighter Si isotopes from the ambient waters [2]. This biased uptake is expected to leave distinct isotopic fingerprints in both biogenic opal and the residual Si-depleted waters, and an increasing number of studies have attempted to use Si stable isotope abundances from marine biogenic materials (e.g., diatoms) and seawater to elucidate marine distribution and cycling of Si [3,4,5]. However, Si is also ‘quasi-essential’ for the growth of higher plants [1], and as a result, researchers have also begun to investigate Si isotope-related processes in terrestrial plants [6].

Terrestrial plants require Si for optimal growth and are also a major component of the global Si cycle [7]. For example, terrestrial plants can accumulate high levels of Si, ranging from 0.1 to 10.0% (dry weight) [1], and plants also contribute to the weathering of silicate rocks by transporting CO_2_ from the atmosphere into the soil, which accelerates the erosion of silicate rocks by forming soluble silicic acid [8]. In addition, the total global uptake of Si by terrestrial plants is estimated at 60–200 Tmol per year [9], which is comparable in magnitude to the fixation of oceanic Si by diatoms (240 Tmol year^-1^) [10].

Recently, researchers have also addressed the composition of Si isotopes in higher plants. For example, the fractionation of Si isotopes has been observed in bamboo [11,12], rice [13,14,15,16], banana [17,18], and wheat [19]; and investigation of Si isotopes in Chinese herbal medicine [20] has provided a more theoretical basis for future research. In addition, Köster et al. [21] conducted a preliminary investigation of Si isotope distribution in the stems and husks of rice and found that the SiO_2_ contents and δ^30^Si values were both lower in Si absorption-defective mutants than in wild-type plants. However, there is no clear explanation for this phenomenon, since the mechanism of Si uptake in rice remains to be determined and because the process of Si circulation in terrestrial plants is still poorly understood, relative to that of marine organisms, such as diatoms [22,23,24].

The literature suggests that two types of kinetic Si isotope fractionation processes occur in plants: one by which Si is absorbed by plant roots and another by which silica is deposited in plant tissues [16]. However, published studies of terrestrial plants have only focused on monocot species and typically only use a single Si concentration [14,15,16]. As a result, analyzing the isotope compositions of “ambient waters” at different Si levels has been impossible. Thus, additional research in higher plants is clearly needed.

As a model plant, most studies of Si isotope fractionation mechanisms have been conducted in rice. Among higher plants, rice is a typical Si accumulator that can accumulate up to 10% SiO_2_ content (dry weight) [1], and the species is also a classic model plant for studying plant physiology, as well as an important food source throughout most of the world. However, investigating isotopic fractionation in rice will also elucidate Si nutrition mechanisms in other crop species and in plants in general. Therefore, in the present study, we examined the isotope fractionation of rice seedlings grown with different external Si concentrations, and we also investigated whether the active and passive mechanisms of Si uptake and transport could function simultaneously.

## Results

**Fig 1:**
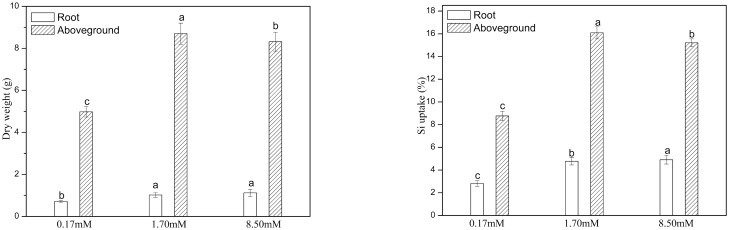
Biomass and measured Si uptake by rice grown with external supply of Si at 0.17 mM, 1.70mM and 8.50mM. Means marked with different letters denote a significant difference at *P* < 0.05.

The biomass of rice plants grown in nutrient solutions with 1.70 and 8.50 mM Si was higher than that of plants grown in nutrient solutions with 0.17 mM Si, and the SiO_2_ content of the above- and belowground plant parts exhibited a similar trend (Fig 1). The root δ^30^Si values of plants supplied with 0.17, 1.70, and 8.50 mM Si were 0.03, 0.15, and 0.19‰, respectively, whereas those of the aboveground parts were lower, with values of -0.31, -0.78, and -1.08‰, respectively (Table 1), and the isotopic composition of rice plantlets was heavier when supplied with lower Si levels and lighter when supplied with higher Si levels. The fractionation of Si isotopes in roots was less exaggerated than that in the aboveground plant parts. In rice plantlets, the average intra-plant fractionation (Δ^30^Si_aboveground-root_) was -0.84 ± 0.07‰, and the degree of fractionation increased with the increased Si level of the nutrient solutions.

**Table 1:** Intra-plant fractionation Δ^30^Si (‰) in rice seedlings for each Si supply (mM) and ^30^ε between the bulked plant δ^30^Si_plant_ (‰) and the source δ^30^Si_source_ (‰).

	Δ^30^Si			^30^ε			
	δ^30^Si_aboveground_	δ^30^Si_root_	Δ^30^Si_aboveground-root_	δ^30^Si_plant_	δ^30^Si_source_	δ^30^Si_solution_	^30^ε_plant-source_
	‰	‰	‰ ± σ_D_	‰	‰	‰	‰ ± σ_D_
8.50 mM	-1.08 ± 0.04c	0.19 ± 0.06a	-1.27 ± 0.10c	-1.03 ± 0.09c	0.10	0.50 ± 0.04a	-1.13 ± 0.09c
1.70 mM	-0.78 ± 0.15b	0.15 ± 0.09ab	-0.94 ± 0.24b	-0.75 ± 0.07b	0.10	0.35 ± 0.02b	-0.85 ± 0.07b
0.17 mM	-0.31 ± 0.06a	0.03 ± 0.08b	-0.34 ± 0.11a	-0.30 ± 0.05a	0.10	-0.36 ± 0.02c	-0.40 ± 0.05a
Average	-0.72 ± 0.05	0.12 ± 0.02	-0.84 ± 0.07	-0.69 ± 0.07	0.10	0.16 ± 0.03	-0.79 ± 0.07

Data are expressed as means ± SD (n = 4). δ^30^Si_solution_ (‰) are expressed as the δ^30^Si values of nutrient solution after Si uptake by plants.

**Fig 2:**
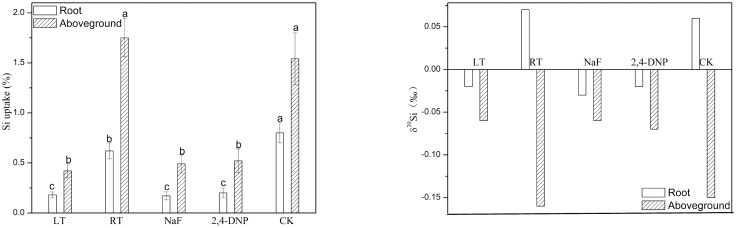
Measured Si uptake and Si isotope composition in rice seedlings treated with low temperature and inhibitors. Means marked with different letters denote a significant difference at *P* <0.05. LT-low temperature, RT-room temperature.

The SiO_2_ contents of rice plants treated with either metabolic inhibitors or low temperature were lower than those of untreated plants (Fig 2), and the isotopic composition of the untreated plants was slightly heavier than that of the nutrient solutions after plant uptake (Table 2). The reduced SiO_2_ contents of the nutrient solutions without either metabolic inhibitors or low temperature were obvious, even though only small amounts of Si were taken up by the plants treated with either metabolic inhibitors or low temperature. The difference of the δ^30^Si values both of the plants and solutions after plant uptake (^30^ε) and of the Si solution source and solutions after plant uptake indicated that the untreated rice seedlings were depleted of ^28^Si, relative to the nutrient solutions (i.e., compared to the nutrient solutions, the rice seedlings were relatively enriched in ^30^Si), whereas treatment with either metabolic inhibitors or low temperature resulted in a large depletion of ^30^Si, relative to the nutrient solutions (i.e., compared to the nutrient solutions, the rice seedlings were relatively enriched in ^28^Si). Thus, greater fractionation of the Si isotopes was observed in the untreated rice seedlings, and the absolute magnitude of the fractionation factor was higher in plants exposed to metabolic inhibitors or low temperature.

**Table 2:** Si concentration and δ^30^Si values of solution and whole rice plant treated with low temperature and inhibitors.

Treatment	Si concentration	δ^30^Si_source_	δ^30^Si_solution_	δ^30^Si_plant_	Δ^30^Si_aboveground-root_	^30^ε_plant-solution_
	mM	‰	‰	‰	‰ ± σ_D_	‰ ± σ_D_
Source nutrient solution	0.17a	0.06	-	-	-	-
Low-temperature (6h)	0.16 ± 0.002a	0.06	0.14 ± 0.02a	-0.06 ± 0.04a	-0.04 ± 0.04a	-0.20 ± 0.04a
Room temperature (6h)	0.13 ± 0.010c	0.06	-0.23 ± 0.04b	-0.15 ± 0.03b	-0.23 ± 0.06b	0.08 ± 0.03b
Inhibitor (6h) NaF	0.16 ± 0.004a	0.06	0.15 ± 0.02a	-0.06 ± 0.03a	-0.03 ± 0.03a	-0.21 ± 0.03a
2, 4-DNP	0.16 ± 0.005a	0.06	0.14 ± 0.03a	-0.07 ± 0.04a	-0.05 ± 0.05a	-0.21 ± 0.04a
CK	0.14 ± 0.005b	0.06	-0.20 ± 0.03ab	-0.13 ± 0.04ab	-0.21 ± 0.08b	0.07 ± 0.04ab

Data are expressed as means ± SD (n = 6). δ^30^Si_solution_ (‰) are expressed as the δ^30^Si values of nutrient solution after Si uptake by plants.

## Discussion

### Plant δ^30^Si at different levels of Si supplementation

When comparing the isotope compositions of rice plants to those of the nutrient solutions after plant uptake, the results suggest that heavy Si isotopes are preferentially absorbed by plants at lower levels of Si supplementation and that light Si isotopes are preferentially absorbed at higher levels of Si supplementation. For example, at the lower Si concentration (i.e., 0.17 mM Si), we observed a slight depletion of ^28^Si in the rice seedlings, relative to the nutrient solutions, after plant uptake, whereas at the higher Si concentration (i.e., 8.50 mM Si), we observed a large depletion of ^30^Si in the rice seedlings. It is well established that carrier-mediated transport would result in the preferential absorption of heavy isotopes, owing to the stronger affinity between heavy isotopes and carrier proteins [16,25,26,27], whereas transport *via* mass-flow and ion channels would result in the preferential absorption of lighter isotopes, owing to greater diffusion coefficients [16,27,28,29]. Thus, based on these data obtained in this study, we speculate that the Si uptake in rice seedlings might be mainly dominated by carrier-mediated transport at lower external Si concentrations, by which active transport systems exhibit a preference for heavy isotopes. While at higher external Si concentrations, the passive transport systems might exhibit a preference for light isotopes.

Previous studies have shown that the mechanisms of Si uptake and transport in rice roots are jointly controlled by three transporters (Lsi1, Lsi2 and Lsi6) [30,31,32], that these transporters are located at nodes, that an apoplastic barrier is present at the bundle sheath cells, and that development of enlarged vascular bundles at nodes are all required for the preferential distribution of Si in aboveground parts [33]. Therefore, active Si uptake obviously occurs in rice. However, passive uptake may also play an important role, especially at higher levels of Si supplementation, which is why we further investigated the specific Si uptake mechanisms of rice seedlings through short-term cultivation.

### Effect of low temperature and metabolic inhibitors on δ^30^Si

The mechanism of active ion uptake or transport across membranes, which is driven by H^+^-ATP pump [34] or ATP-binding cassette transporters [35], is often believed to be an ‘uphill’ process that is opposed by the electrochemical potential gradient and that is characterized by high selectivity in ion uptake and energy consumption. The metabolic inhibitor 2,4-DNP uncouples oxidative phosphorylation, photosynthesis (to a lesser extent), and proton-coupled fluxes at both the plasma membrane and endomembranes, and the inhibitor also exerts its effect by dissipating the transmembrane electrochemical gradients of protons [36,37,38,39]. In contrast, NaF primarily inhibits the process of glycolysis [36,39], thus inhibiting the formation of ATP. This cannot provide energy for active ion uptake or transport and further inhibits the formation of ATP-binding cassette transporters. Meanwhile, low temperature can affect the activity of ATP-binding cassette transporters. Therefore, these compounds can affect Si acquisition either directly or indirectly.

The observed reduction in Si acquisition by the either metabolic inhibitors or low temperature is consistent with Si uptake being an ‘uphill’ process that is opposed by the electrochemical potential gradient, driven by the H^+^-ATP pump or ATP-binding cassette transporters, and characterized by energy-consuming mechanisms. Therefore, active uptake of Si should not be neglected completely. However, it is also important to recognize that the level of active uptake was reduced significantly by treatment with low temperature and metabolic inhibitors and that these weak effects of active Si uptake can be ignored under these conditions, especially at low external Si concentrations (e.g., 0.17 mM). The SiO_2_ content absorbed by untreated plant roots was about three times higher than that absorbed by plants under either low temperature or metabolic inhibitor treatment. This suggests that the passive mechanism is only responsible for a small proportion of the overall Si uptake at lower levels of external Si.

Opfergelt et al. [18] reported that the roots of hydroponically grown banana seedlings were enriched in heavier Si isotopes, compared to aboveground parts, which suggested that two fractionation mechanisms were involved. That is, the preferential transport of light Si isotopes exists in both the endodermis and epidermis of roots. These Si isotopes transferred by the mechanism of xylem-loading to the aboveground plant parts is depletion of ^30^Si (rich in ^28^Si), relative to the external solutions entering the roots. Therefore, Si residing in the roots is isotopically heavier than that in the aboveground plant parts.

In the present study, the roots of rice plants that were treated with either low temperature or metabolic inhibitors also had higher relative levels of ^30^Si than the aboveground parts. To some extent, we assume that the explanation proposed by Opfergelt et al. [18] is compatible with our findings. However, unlike the roots of banana seedlings, the Si isotope compositions of rice roots appear to reflect the mixing of two components: depositional Si (opal) and dissolved Si (monomeric silicic acid). According to the respective proportions of depositional and dissolved Si in our previous studies, we conclude that the dissolved Si in the roots of seedlings at the early stage of growth represented >40% of the overall root Si budget and, therefore, a significant proportion of the total Si. In addition, the isotope composition of the dissolved Si was much heavier.

On the other hand, because early-stage rice seedlings are very small, with few and underdeveloped leaves and with imperfectly developed and relatively few nodes, the transporters located in the leaves and nodes that preferentially incorporate heavier Si isotopes might be incompletely expressed, which would result in a weaker active Si uptake mechanism in the aboveground parts. Furthermore, according to the principle of kinetic isotope fractionation, as dissolved H_4_^28^SiO_4_ preferentially precipitates to form biogenic opal during transpiration [40], so that the preferential transfer of light Si isotopes to the aboveground parts along with the transpiration might also play a very important role. Generally, Si is rapidly absorbed and transported in untreated plants. Thus, more light Si isotopes may be transported from the roots to the aboveground parts along with the action of the transpiration stream, resulting in a lighter isotopic composition in untreated aboveground parts than in aboveground parts treated with either low temperature or metabolic inhibitors. In addition, given that the low-temperature and inhibitor experiments were conducted in closed systems and that the system had no external Si input, the isotopic composition of the nutrient solutions may be closely related to that of the whole plants. For example, when external nutrient solutions are relatively heavy, the isotopic composition of whole plants may also be heavier.

### Silicon isotope fractionation

In the present study, it seems that the content and contribution of dissolved Si to the δ^30^Si values of the roots became progressively smaller as the Si concentration of the nutrient solution decreased, and conversely, the δ^30^Si values of the roots became larger as the Si concentration increased. We hypothesize that there is a close relationship between the levels of external Si supplementation and the dissolved Si content present in the rice roots. However, it is impossible to prove this assertion from the data collected, since the root opal was not separated from the dissolved Si in the roots.

The nutrient solutions with Si levels of 0.17 and 1.70 mM were replaced frequently during the 36 d after transplanting the seedlings, in order to maintain stable Si concentrations. However, the frequent replacement might have also caused fluctuations in the Si isotopic composition, due to differences in the batches of nutrient solutions. Although the nutrient solutions with 8.50 mM Si were never replaced over the 36 d, the systems still received no external Si input, and the system of low-temperature and inhibitor experiments also received no external Si input. Therefore, the treatments in which the solution was replaced can be considered examples of conventional cultivation, which is an open system, whereas the unreplenished solution represents a hydroponic model, which is a closed system, with a limited source of Si. According to Varela et al. [41], the fractionation factor (^30^ε) of open systems should be calculated as the difference between the biogenic silica (phytoliths) and dissolved silicic acid in the initial nutrient solutions, which could make the calculation process have a consistent standard, whereas that of the closed system should be calculated as the difference between the biogenic silica (phytoliths) and dissolved silicic acid in nutrient solutions after plant uptake. In the present study, the Si-uptake experiments at different Si supplied levels were regarded as open systems, and the low-temperature and inhibitor experiments were regarded as the closed systems.

Since the difference of ^30^ε between the lowest and the highest Si concentrations was relatively large (0.73‰) and the difference between the low-temperature or metabolic inhibitor treatments and the control treatment was relatively small (0.29‰), we cannot rule out the influence of Si supply to the variation of fractionation factors. In the low-temperature and inhibitor experiments, the biological processes of fractionation could be explained by the fact that both active and passive Si uptake components co-exist in rice, and the fractionation effect will be enhanced when more Si is absorbed by plants. There is a general trend towards larger absolute values (^30^ε) in response to greater Si concentrations, and the difference between ^30^ε and Δ^30^Si was greater in the control rice seedlings, i.e., those no treated with either metabolic inhibitors or low temperature (Table 2). For smaller Si isotopic fractionation, Δ^30^Si can be used to approximate ^30^ε, if an isotopic equilibrium is achieved between the dissolved and particulate phases [2,18,41,42]. Therefore, the fractionation effect of the uptake mechanisms might be enhanced when the ambient environment provides more Si nutrition. In the present study, the biological processes of Si isotope fractionation occurred when the rice plants absorbed Si from nutrient solutions through their roots. Similar fractionation has been reported in hydroponically grown wheat, banana, and rice [15,18,43]. Compared with the ^30^ε of other plant species (Table 3), the fractionation factor of the rice seedlings in the present study (^30^ε = -0.79 ± 0.07‰) is very similar to that calculated for hydroponically grown banana (^30^ε = -0.77 ± 0.21‰) [18] and is smaller in absolute magnitude than that calculated for field-grown rice (^30^ε = -1.02 ± 0.33‰) [13] and hydroponically grown wheat and corn (^30^ε = -1.00 ± 0.31‰) [43]. These differences in fractionation factors could reflect, at least in part, the strength of Si fractionation and the uptake mechanism of Si during plant Si uptake in the biological processes. Thus, Si isotopic fractionation factors vary among different plant species, and their extent might be influenced by physiological and environmental conditions, including Si availability [44].

**Table 3:** Si isotope compositions of different plants.

	Plant	δ^30^Si_plant_	δ^30^Si_source_	^30^ε_plant-source_
		‰	‰	‰ ± σ_D_
Ziegler et al. (2005)	Corn, Wheat^a^	-1.20	-0.19	-1.00 ± 0.31
Opfergelt et al. (2006)	Banana^a^	-0.66	0.12	-0.77 ± 0.21
Ding et al. (2005)	Rice	-0.02	1.00	-1.02 ± 0.33
This study	Rice^a^	-0.69	0.10	-0.79 ± 0.07

δ^29^Si data were conver to δ^30^Si by a division factor of 1.93.

^a^Hydroponics.

Indeed, the distribution of Si among plant parts could also be affected by additional processes. For example, some organic compounds (e.g., hydroxyl-containing organic molecules and polyamines) have been reported to affect the progression of Si deposition in rice plants and diatoms [45,46,47]. Studies have also shown that the enrichment of plants with heavy Mg isotopes is associated with organic acids in the plants’ roots, and even the slight enrichment of light Mg isotopes in aboveground parts has been demonstrated to affect biological processes, including the formation of organic molecules [48]. In addition, biological factors also have a significant effect on the δD_*n*-alkane_ values of leaf wax in higher plants [49]. Therefore, biological factors might have a significant impact on the fractionation of other nutrients, as well.

### Potential applications of Si isotope research

Because opaline silica is quite stable in soil environments, phytoliths are preserved in soil environments after plant death. Thus, phytoliths may serve as a useful indicator of the paleoclimate. Recent research has also indicated that the absorption of Si by rice is closely associated with C sequestration, since C can be occluded in phytoliths (i.e., PhytOC) for long periods of time, and the PhytOC content of cultivated rice can be increased considerably by augmenting Si nutrition (e.g., Si fertilizers and basalt powder amendment) [50,51], in order to increase C sequestration. These findings may significantly influence efforts to manage the global C cycle by reducing the concentration of greenhouse gasses in the atmosphere [50] and indirectly mitigating climate change [51]. Furthermore, Li et al. [52] report that the solubility of rice phytoliths in soils may be affected by cultivar and organ type. For example, compared to rice grain and stems, the solubility of phytoliths from rice leaves and sheaths is higher. Therefore, the use of different rice cultivars and plant organs as soil amendment plays an important role in the release of dissolved Si in the soil, which can further affect the subsequent absorption of soluble silica by other plants.

In addition, the composition of Si isotopes in different plant cultivars and organs should also be considered when using the Si isotope composition of phytoliths from terrestrial plants as palaeoecological or archaeological proxies. Future investigations should focus on Si isotope fractionation during xylem loading and the radial movement of Si in other Si-accumulating dicots, such as cucumber and sunflower. The Si uptake and transport mechanisms of higher plants are complex and appear to have an impact on the Si isotope fractionation of terrestrial ecosystems. Therefore, further studies are needed to determine whether Si uptake and transport mechanisms are also associated with other factors.

## Methods

### Plant materials and growth conditions

Pot experiments were conducted in a glasshouse at Zhejiang University, Hangzhou, China in 2012, using rice (*Oryza sativa* L. ‘Zhenong 952’). Uniform-sized seeds were surface-sterilized using 10% (v/v) H_2_O_2_ for 5 min and then rinsed 10 times with deionized water to wash away residual H_2_O_2_. The sterilized rice seeds were germinated on a 20% (w/v) HCl-treated sand bed at 25°C in the dark, and after 25 d, uniform-sized seedlings were transferred to black plastic-covered buckets (28 cm diameter; two seedlings per pot) that each contained 10 L nutrient solution [53]. The nutrient solution was aerated daily in order to improve gas exchange by the roots. It is widely accepted that Si is absorbed by plant roots in the form of silicic acid as an uncharged monomeric molecule. The monomeric silica was steady-state at pH 6.0 approximately in the equilibrium solution [54]. Thus in the present study, Si was supplied *via* Na_2_SiO_3_ 9H_2_O (analytical reagent) that had been neutralized with dilute H_2_SO_4_ before use [20], and the pH of the nutrient solutions was adjusted to ~6.0 on a daily basis, using 10 mM H_2_SO_4_.

#### Si-uptake experiments

Plants were exposed to three Si levels: 0.17, 1.70, and 8.50 mM. Deionized water was added each day, in order to replenish the amount of water lost *via* transpiration, and the major nutrients N, P, K, Mg, and Ca were replenished each week using NH_4_NO_3_, K_2_SO_4_, NaH_2_PO_4_, MgSO_4_, and CaCl_2_, in order to avoid nutrient imbalance or depletion. In order to maintain constant levels of Si in the nutrient solutions, when the seedlings reached the tillering stage, the nutrient solutions with 0.17 mM Si were changed once each 4 d for 12 d, once each 3 d for the following 9 d, and once each 2 d for the following 14 d, whereas the nutrient solutions with 1.70 mM Si were changed once after 15 d and once each 7 d for the following 21 d. Meanwhile, the nutrient solutions with 8.50 mM Si were not changed or replenished. Each experiment was repeated four times.

At 36 d after the seedlings were transferred to the buckets, the roots and aboveground parts of the plant samples were processed separately. The roots and aboveground parts were both rinsed three times (5 min each) in a plastic bucket that contained 5 L deionized water, in order to remove surface bound nutrients. Afterward, the samples (both the roots and aboveground parts) were oven-dried at 80°C for 72 h. The dry weight of each plant part was measured, and then the dried material was ground through a 0.5 mm sieve and stored for further analysis.

#### Low-temperature and inhibitor experiments

For low-temperature treatment, 20-d-old rice seedlings were exposed to nutrient solutions with 0.17 mM Si. The nutrient solutions were precooled and maintained at 4°C before starting the experiment, and the low temperature was maintained for 6 h using an ice-bath. In order to examine the effect of low temperature on the subsequent Si uptake, the ice-bath was removed, and the temperature of the nutrient solutions was allowed to progressively increase to room temperature (25°C) over 10 h, after which the experiment was allowed to continue at 25°C for another 6 h. Meanwhile, to examine the effect of metabolic inhibitors on Si uptake, 20-d-old rice seedlings were also exposed to nutrient solutions with 0.17 mM Si and either 1.0 mM NaF or 0.1 mM 2,4-dinitrophenol (2,4-DNP) for 6 h. The 2,4-DNP was dissolved in ethanol, and in order to maintain consistency, the same concentration of ethanol was also added to the control treatment solution.

For both experiments, each uniform-sized plant was grown in 50 ml treatment. These plants were washed as described in the previous section. At the end of the experiments, the plants were separated into the roots and aboveground parts, and the dry weights of each part were recorded after being oven-dried at 80°C for 72 h. All the experiments were repeated six times, and the ground plant material was stored for further analysis.

### Measurements

The Si and SiO_2_ contents were extracted from the above- and belowground plant parts and the nutrient solutions, respectively, as described previously [55], and were measured using the colorimetric molybdenum blue method at 700 nm [54].

Meanwhile, the Si isotope compositions of the above- and belowground plant parts and the nutrient solutions were measured using multi-collector inductively coupled plasma mass spectrometry (MC-ICP-MS), as described previously [56]. Briefly, 5 mg of each sample was mixed with 60 mg ultrapure solid sodium hydroxide monohydrate in a 7 ml perfluoroalkoxy beaker (Savillex, Eden Prairie, MN, USA), and the mixture was decomposed in a high-pressure digester (Parr bomb; Parr Instrument Co., Moline, IL, USA) at 200°C for 3 d. After cooling, the sample was mixed with 2 ml ultrapure water and then heated on a hot plate at 100°C for 2 h. If the mixture produced a brownish precipitate, the mixture was transferred to a 5 ml centrifuge tube and centrifuged at 11,000 rpm for 4 min. Then, the supernatant was removed, and the precipitate was dissolved by adding 100 μl 7 M HNO_3_ and heating the mixture to 100°C for at least 5 h in a sealed system. The dissolved Si was exchanged using cation-specific exchange resin (AG50-X8; Bio-Rad Laboratories, Hercules, CA, USA), and the Si concentrations of the final solutions were adjusted to 2 μg g^-1^. Finally, the standard solutions and samples were measured using a Neptune MC-ICP-MS (Thermo Fisher Scientific, Waltham, MA, USA) in high-resolution (HR) mode (Rpower = 4300), and the δ^30^Si values of each sample were calculated using (Eq 1):

$$\delta^{30}\text{Si}\left(‰\right)=\left[\left({\text{R}}_{\text{sam}}/{\text{R}}_{\text{std}}\right)-1\right]\times 10^{3}$$ (1)

where *R*_sam_ and *R*_std_ are the ^30^Si/^28^Si ratios of the sample and NIST SRM 8546 (National Institute of Standard and Technology RM #8546, formerly NBS-28), respectively. The total analytical precision was determined by repeated determination of two Chinese national reference materials for Si isotopes, GBW04421 and GBW04422.

The intra-plant fractionation (Δ^30^Si_root-aboveground_, ‰) between the roots and aboveground plant parts was estimated as the difference between the corresponding δ^30^Si values (δ^30^Si_root_ and δ^30^Si_aboveground_), using (Eq 2):

$$\Delta^{30}{\text{Si}}_{\text{root}-\text{aboveground}}=\delta^{30}{\text{Si}}_{\text{aboveground}}-\delta^{30}{\text{Si}}_{\text{root}}$$ (2)

In addition, the fractionation of Si isotopes by bio-fractionation processes was measured as the fractionation factor (^30^ε, ‰) between the biogenic silica (phytoliths) and dissolved silicic acid (nutrient solutions), which in the open system was estimated using (Eq 3) and in the closed system was estimated using (Eq 4):

ε30~Δ30Si=δ30Siplant−δ30Sisource (3)

ε30~Δ30Si=δ30Siplant−δ30Sisolution (4)

where δ^30^Si_plant_ represents the δ^30^Si values of whole plants and δ^30^Si_source_ and δ^30^Si_solution_ represent the δ^30^Si values of initial nutrient solutions and nutrient solutions after plant uptake, respectively.

### Statistical analysis

All experimental data reported were average means ± standard deviation (SD) with four replicates each. The statistical significance (*P* < 0.05) of differences observed among the means of treatment groups was tested using Duncan’s new multiple range tests in SPSS 13.0 for Windows (SPSS, Chicago, IL, USA).

## Supporting Information

S1 Fig Biomass and measured Si uptake of root and aboveground part at different Si supplied levels. (DOCX)

S2 Fig Biomass, measured Si uptake and δ^30^Si value of root and aboveground part treated with low temperature and inhibitors. (DOCX)

S1 Table δ^30^Si value of root, aboveground part, source nutrient solution, and nutrient solution after plant uptake at different Si supplied levels. (DOCX)

S2 Table Si concentration and δ^30^Si value of source nutrient solution and nutrient solution after plant uptake treated with low temperature and inhibitors. (DOCX)
