# Supplementary material for: Experimental Determination of Silicon Isotope Fractionation in Rice
Source: PLoS One. 2016 Dec 30;11(12):e0168970. doi: 10.1371/journal.pone.0168970 (PMC5201238; doi:10.1371/journal.pone.0168970)
Supplement: S1 Fig — (DOCX) [file pone.0168970.s001.docx]

| Dry weight (g) | Root | | | Aboveground | | |
| --- | --- | --- | --- | --- | --- | --- |
| N | 0.17mM | 1.70mM | 8.50mM | 0.17mM | 1.70mM | 8.50mM |
| 1 | 0.70 | 1.02 | 1.12 | 5.05 | 9.26 | 8.41 |
| 2 | 0.77 | 1.17 | 1.05 | 4.76 | 8.61 | 7.75 |
| 3 | 0.63 | 0.85 | 0.93 | 5.28 | 8.09 | 8.89 |
| 4 | 0.73 | 1.05 | 1.36 | 4.82 | 8.85 | 8.22 |

| Si uptake (%) | Root | | | Aboveground | | |
| --- | --- | --- | --- | --- | --- | --- |
| N | 0.17mM | 1.70mM | 8.50mM | 0.17mM | 1.70mM | 8.50mM |
| 1 | 2.85 | 4.82 | 4.95 | 8.83 | 16.15 | 15.26 |
| 2 | 3.12 | 4.40 | 5.35 | 8.27 | 15.52 | 15.62 |
| 3 | 2.51 | 5.19 | 4.46 | 9.26 | 16.80 | 14.87 |
| 4 | 2.78 | 4.71 | 4.86 | 8.70 | 15.87 | 15.07 |
