# Supplementary material for: Experimental Determination of Silicon Isotope Fractionation in Rice
Source: PLoS One. 2016 Dec 30;11(12):e0168970. doi: 10.1371/journal.pone.0168970 (PMC5201238; doi:10.1371/journal.pone.0168970)
Supplement: S2 Fig — (DOCX) [file pone.0168970.s002.docx]

| Si uptake (%) | Root | | | | | Aboveground | | | | |
| --- | --- | --- | --- | --- | --- | --- | --- | --- | --- | --- |
| N | LT | RT | NaF | 2,4-DNP | CK | LT | RT | NaF | 2,4-DNP | CK |
| 1 | 0.14 | 0.58 | 0.21 | 0.27 | 0.94 | 0.32 | 1.66 | 0.53 | 0.68 | 1.56 |
| 2 | 0.18 | 0.73 | 0.12 | 0.20 | 0.75 | 0.48 | 2.03 | 0.38 | 0.41 | 1.86 |
| 3 | 0.20 | 0.55 | 0.15 | 0.15 | 0.72 | 0.45 | 1.61 | 0.58 | 0.47 | 1.22 |
| 4 | 0.21 | 0.60 | 0.20 | 0.17 | 0.80 | 0.41 | 1.70 | 0.45 | 0.50 | 1.50 |

LT-low temperature, RT-room temperature.

| δ^30^Si (‰) | Root | | | | | Aboveground | | | | |
| --- | --- | --- | --- | --- | --- | --- | --- | --- | --- | --- |
| N | LT | RT | NaF | 2,4-DNP | CK | LT | RT | NaF | 2,4-DNP | CK |
| 1 | -0.03 | 0.08 | -0.03 | -0.05 | 0.09 | 0.00 | -0.12 | -0.09 | -0.13 | -0.13 |
| 2 | -0.04 | 0.03 | -0.07 | -0.03 | 0.03 | -0.12 | -0.22 | -0.03 | -0.06 | -0.21 |
| 3 | 0.02 | 0.10 | 0.01 | 0.00 | 0.07 | -0.07 | -0.15 | -0.05 | -0.03 | -0.15 |
| 4 | -0.04 | 0.05 | -0.01 | 0.02 | 0.05 | -0.04 | -0.14 | -0.06 | -0.07 | -0.09 |

LT-low temperature, RT-room temperature.

| Dry weight (g) | Root | | | | | Aboveground | | | | |
| --- | --- | --- | --- | --- | --- | --- | --- | --- | --- | --- |
| N | LT | RT | NaF | 2,4-DNP | CK | LT | RT | NaF | 2,4-DNP | CK |
| 1 | 0.013 | 0.008 | 0.015 | 0.019 | 0.013 | 0.079 | 0.076 | 0.081 | 0.084 | 0.093 |
| 2 | 0.010 | 0.007 | 0.009 | 0.014 | 0.019 | 0.089 | 0.098 | 0.092 | 0.093 | 0.099 |
| 3 | 0.014 | 0.015 | 0.012 | 0.013 | 0.010 | 0.076 | 0.085 | 0.071 | 0.074 | 0.081 |
| 4 | 0.016 | 0.011 | 0.014 | 0.012 | 0.012 | 0.071 | 0.095 | 0.080 | 0.086 | 0.089 |

LT-low temperature, RT-room temperature.
