# Supplementary material for: Experimental Determination of Silicon Isotope Fractionation in Rice
Source: PLoS One. 2016 Dec 30;11(12):e0168970. doi: 10.1371/journal.pone.0168970 (PMC5201238; doi:10.1371/journal.pone.0168970)
Supplement: S1 Table — (DOCX) [file pone.0168970.s003.docx]

| δ^30^Si (‰) | Root | | | Aboveground | | |
| --- | --- | --- | --- | --- | --- | --- |
| N | 8.50mM | 1.70mM | 0.17mM | 8.50mM | 1.70mM | 0.17mM |
| 1 | 0.26 | 0.18 | 0.00 | -1.12 | -0.74 | -0.38 |
| 2 | 0.13 | 0.05 | 0.13 | -1.04 | -0.67 | -0.33 |
| 3 | 0.20 | 0.25 | -0.05 | -1.07 | -1.00 | -0.28 |
| 4 | 0.16 | 0.12 | 0.04 | -1.10 | -0.70 | -0.23 |

| δ^30^Si (‰) | Source | | | Solution | | |
| --- | --- | --- | --- | --- | --- | --- |
| N | 8.50mM | 1.70mM | 0.17mM | 8.50mM | 1.70mM | 0.17mM |
| 1 | 0.05 | 0.09 | 0.06 | 0.44 | 0.33 | -0.38 |
| 2 | 0.13 | 0.11 | 0.1 | 0.52 | 0.38 | -0.34 |
| 3 | 0.12 | 0.13 | 0.14 | 0.54 | 0.36 | -0.35 |
| 4 | 0.1 | 0.05 | 0.08 | 0.49 | 0.34 | -0.36 |

δ^30^Si_solution_ (‰) are expressed as the δ^30^Si values of nutrient solution after Si uptake by plants.
