# Supplementary material for: Experimental Determination of Silicon Isotope Fractionation in Rice
Source: PLoS One. 2016 Dec 30;11(12):e0168970. doi: 10.1371/journal.pone.0168970 (PMC5201238; doi:10.1371/journal.pone.0168970)
Supplement: S2 Table — (DOCX) [file pone.0168970.s004.docx]

| δ^30^Si (‰) | Source | | | | | Solution | | | | |
| --- | --- | --- | --- | --- | --- | --- | --- | --- | --- | --- |
| N | LT | RT | NaF | 2,4-DNP | CK | LT | RT | NaF | 2,4-DNP | CK |
| 1 | 0.08 | | | | | 0.15 | -0.20 | 0.13 | 0.10 | -0.24 |
| 2 | 0.02 | | | | | 0.17 | -0.25 | 0.18 | 0.18 | -0.16 |
| 3 | 0.05 | | | | | 0.13 | -0.18 | 0.15 | 0.15 | -0.19 |
| 4 | 0.1 | | | | | 0.12 | -0.27 | 0.13 | 0.12 | -0.22 |

LT-low temperature, RT-room temperature. δ^30^Si_solution_ (‰) are expressed as the δ^30^Si values of nutrient solution after Si uptake by plants.

| Si concentration  (mM) | | | | | |
| --- | --- | --- | --- | --- | --- |
|  | LT | RT | NaF | 2,4-DNP | CK |
| Source | 0.17 | | | | |
| 1 | 0.160 | 0.142 | 0.160 | 0.165 | 0.144 |
| 2 | 0.161 | 0.128 | 0.164 | 0.153 | 0.132 |
| 3 | 0.158 | 0.119 | 0.154 | 0.160 | 0.140 |
| 4 | 0.156 | 0.125 | 0.158 | 0.157 | 0.136 |

LT-low temperature, RT-room temperature.
